# High-Speed Dental Instruments: An Investigation of Protein-Contaminated Dental Handpieces with the Bicinchoninic Acid Assay in Dental Offices in Styria, Austria

**DOI:** 10.3390/ijerph20031670

**Published:** 2023-01-17

**Authors:** Michael Schalli, Birgit Kogler, Tillo Miorini, Michael Gehrer, Franz F. Reinthaler

**Affiliations:** 1 Department for Water-Hygiene and Micro-Ecology, D&R Institute of Hygiene, Microbiology and Environmental Medicine, Medical University of Graz, 8010 Graz, Austria; 2 Institute for Applied Hygiene, 8045 Graz, Austria; 3 Styrian Hospital Corporation KAGes, Hospital Leoben, 8700 Leoben, Austria

**Keywords:** dental instruments, disinfection, decontamination, bicinchoninic acid, protein detection

## Abstract

Due to permanent contact with bodily secretions such as blood and saliva, the dental workplace poses a high risk of infection for patients as well as for personnel. High-speed dental instruments are still considered one of the major hygienic risks, as the high-speed rotation of the attachments leads to the retraction of infectious material from patients’ oral cavities. The aim of this study was to investigate the extent to which dental handpieces are contaminated after use. Spray-water samples were taken from different handpieces used in seven dental offices and protein concentrations were measured photometrically. In the first part of the study, samples were collected from each handpiece before and after the treatment of the patients. Additionally, the changes in protein concentration after consecutive treatments in which the same high-speed dental instrument was used were investigated. The results demonstrated measurable protein concentrations in 91.2% of a total of 398 samples, and 96.4% of the spray-water samples taken after treatment showed a discrepancy from the initial measured protein concentration. In 68.4% an increase in protein concentration was observed, whereas in 27.9% a decrease was measured. In conclusion, the internal contamination of high-speed dental instruments frequently occurs in daily usage and consequently may lead to the transmission of infectious agents by flushing the contaminated water out of the spray water tubes. Moreover, it must be pointed out that internal cleansing of handpieces is insufficient and that a final mechanical disinfection is indispensable.

## 1. Introduction

**Figure 1:**
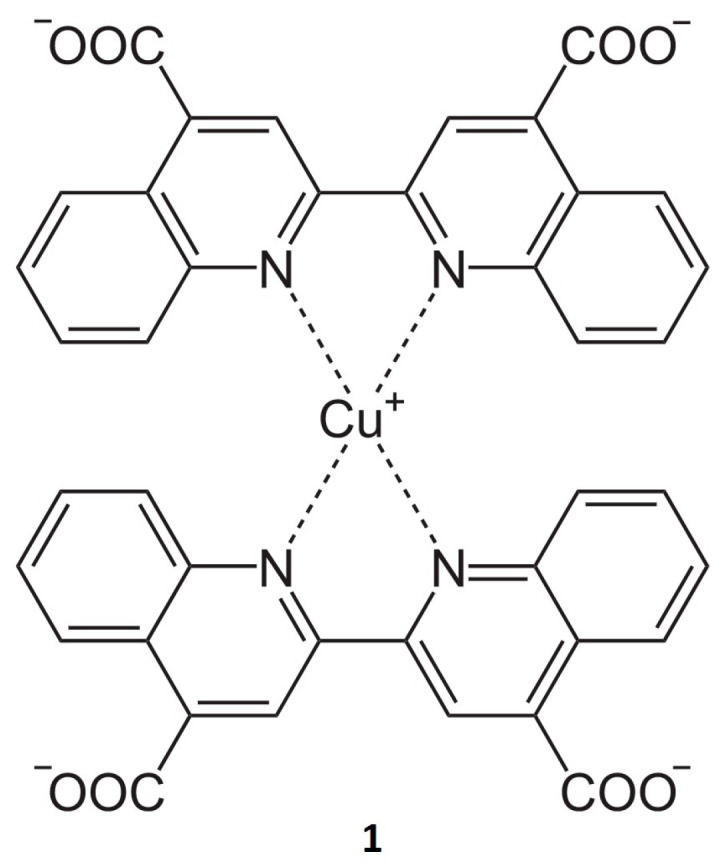
Chelation complex (**1**) containing a Cu^1+^ cation and two molecules of bicinchoninic acid.

Microbial contamination of dental units is a widespread problem in dental medicine and was investigated intensively in the last two decades [1,2,3,4,5,6,7,8,9,10,11,12]. In recently published studies, dental unit waterlines, which provide water for handpieces, water syringes, and mouth-rinse water outlets, were highly contaminated with bacteria [8]. Antibiotic-resistant bacteria (e.g., *Staphylococcus aureus*) were found in 50% of collected samples in a government dental college in a study by Alkhulaifi et al. in 2017 [4]. For this reason, disinfection and sterilization procedures are of common interest in terms of public health. Methods such as ultrasonic cleaning, thermal disinfection, and plasma sterilization were developed for application in dental offices [1,2,3,4,5,6]. The risk of aerosol transmission of pathogenic agents during dental treatment is not fully understood. Dentists use high-speed equipment such as drills and scalers in the presence of bodily fluids such as blood, saliva, and dental plaque [13]. This combination has been shown to generate aerosols of oral micro-organisms and blood [14]. The potential for contamination of rotary dental instruments has received continued attention from investigators [15,16,17,18,19,20]. The models used to assess the risk of contamination of dental handpieces include the uptake of dye-colored water into handpieces [15]. Others show viral DNA in samples taken from inside the equipment and from the attached air/water lines after handpieces have been operated in contact with blood pooled from infected patients [16,21]. The main causes of internal contamination of dental handpieces have been shown to be retraction from the patient’s oral cavity during treatment as well as biofilm formation in the dental unit waterline system [22]. Different approaches for microbial detection in dental unit water systems have been investigated in the past. Aerosols produced by high-speed dental instruments, as well as dental unit-derived liquids containing proteins and microorganisms were examined [23,24]. In 2021, Pasini et al. investigated the impact of self-ligating orthodontic brackets on the formation of dental biofilms in adolescents [25]. The cultivation of bacteria and fungi on different culture media can be a difficult task [26,27]. Different procedures with cultivation times of 24 h to 10 days can be applied to achieve significant results [27,28]. Due to dense timetables in dental offices, methods with long investigation periods can be challenging because results for contamination levels are required quickly and also need to be precise. A method for rapid detection of protein concentration levels can circumvent this problem. Several researchers have developed different methods to detect protein concentration levels in clinical instruments. For example, in one study, eluates from high-speed turbines and low-speed spray channels were analyzed by SDS-PAGE, mass spectroscopy, Western blotting, and ELISA [29]. Büchter and Kruse-Loesler investigated a strategy for protein detection in dental transmission instruments. In their validation study, the Biuret method according to DIN EN 15883-1:2006 was used to determine the residue protein concentrations in channels of transmission instruments [30,31]. In 2014, a study concerning protein quantification in white wine and grape juice used the bicinchoninic acid assay (BCA assay), which was found to have better results than the Bradford assay [32]. The BCA assay was chosen for the determination of protein concentrations in this study. The sodium salt of bicinchoninic acid, a water-soluble organic compound, is capable of forming complexes with copper ions (Cu^1+^) in alkaline solutions. A temperature-dependent reaction, which reduces Cu^2+^ ions to Cu^1+^ ions in the presence of a peptide bond, is followed by the formation of a chelation complex containing one Cu^1+^ ion complexing with two molecules of bicinchoninic acid shown in Figure 1. This purple-colored complex absorbs light at the wavelength of 562 nm, which can be measured with a photometer [33].

Given the described findings, the aim of this study was to determine the extent of contamination of dental instruments after their application by testing spray-water sample protein concentrations using photometry.

## 2. Materials and Methods

Dental units of seven dental offices in Graz, the capital of the Austrian province of Styria, were examined for protein concentrations.

### 2.1. General Procedure for BCA Assay

**Figure 2:**
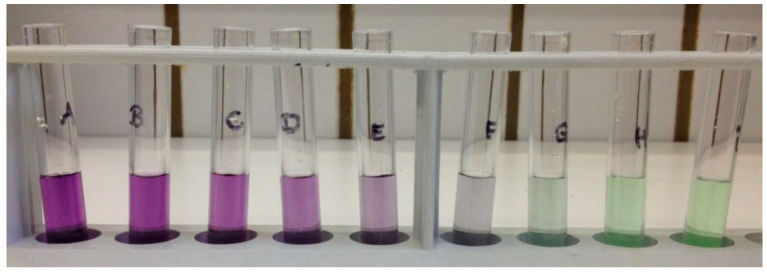
Test tube protocol with a working range of 20–2000 µg/mL (dilution series).

For the preparation of the Standard Test Tube Protocol, a Pierce™ BCA Protein Assay Kit (Thermo Fisher Scientific Inc., Waltham, MA, USA) containing two reagents (Reagent A and Reagent B) and Bovine Serum Albumin (BSA) was used. A dilution series (Figure 2) with a defined BSA concentration was set up according to the manufacturer’s instructions to obtain a standard for the following measurements (shown in Table 1 and Table 2). After preparation of the solutions, the working reagent (WR) was prepared by mixing A and B in a ratio of 50:1 (50 mL A and 1 mL B) in an Erlenmeyer flask for 5 min yielding a clear green solution. The WR (2 mL) was subsequently transferred into a test tube after adding 0.1 mL of standard solution or a spray-water sample. After mixing for 20 s, the samples were incubated at 37 °C for 30 min (for the enhanced test tube protocol, the temperature was 60 °C). After cooling the samples for two minutes to room temperature, the photometric detection was subsequently carried out with a Hitachi U2001 Spectrophotometer (Methrom^®^ Inula GmbH, Vienna, Austria) at 562 nm.

### 2.2. Water Sample Collection

A total of 398 spray-water samples were taken in seven dental offices on working days in the city of Graz, Austria; this is a representative number of samples for protein analysis. Due to the fact that treatments in dental offices stick to a tight schedule, spray-water sample collection was limited to approximately 400 samples. Spray water (1 mL) was collected from the dental handpieces before and after treatment using a sterile tube by starting the engine. Samples were transported in a cool box to the laboratory for further protein analysis.

All dental treatments were limited to conservative dental measures and were carried out using dental handpieces of varying maximum rotational speeds. Red-marked handpieces reached 2 × 10^5^ rpm and were used in every dental office. Five offices also worked with blue and green marked handpieces with a maximum rotational speed of 4 × 10^4^ rpm (blue) and 2 × 10^4^ rpm (green), respectively. Handpiece turbines that run at 4 × 10^5^ rpm were used in only two dental offices (Table 3). To show the possible contamination of the internal surfaces after handpiece usage, one milliliter of spray water was collected as a reference from the decontaminated handpieces before application. Decontamination procedures differed in the participating offices (Table 4). In five out of the seven offices, the external surface of the handpiece was wiped with a disinfectant after each usage. A mechanical decontamination procedure was performed at the end of the working day. One office only wiped the instruments with a disinfectant after each treatment, and one office performed a decontamination at the end of the working day consisting of mechanical cleansing, mechanical disinfection, and final sterilization in addition to wiping the instruments with a disinfectant after every use. All instruments were stored dust free and unpacked immediately before they were fixed at the dental unit’s lugs. To determine the degree of contamination of a high-speed instrument’s internal surface right after contact with blood and saliva, a one-milliliter spray-water sample was collected in a sterilized tube by restarting the engine. After following dental treatment and external disinfection, the samples were all marked with the corresponding reference sample.

Trial series A

The goal of this trial series was to show the differing protein concentrations of samples before and after treatment. In general, the instruments were used more than once a day, and several samples were compared to the initial reference sample before considering the increase or decrease compared to the previous sample. The latter was examined as part of test series two.

Trial series B

In order to detect a possible trend of concentration increase or decrease, three dental treatments each were performed in offices two and seven using the same handpiece on two consecutive days wiping with a disinfectant after each treatment and mechanical cleansing at the end of the first day. The sampling procedure was identical to that in test series one.

### 2.3. Data Analysis

The regression analysis for both test tube protocols was performed with the program SPSS (IBM, Armonk, NY, USA). For the test tube protocol with a working range of 20–2000 µg/mL, a quadratic regression model (Equation (1)) was used, whereas for the enhanced test tube protocol with a working range of 5–250 µg/mL, a linear regression model was chosen (Equation (2)).

## 3. Results

The BCA assay was used to quantify the protein contamination of the spray water. This method is based on the reduction of Cu^2+^ to Cu^1+^ by the aromatic tyrosine and tryptophan side chains of proteins and is less susceptible to interfering compounds than the Lowry method [33,34,35]. The detection range is between 5 μg/mL and 2000 μg/mL. After subsequent photometry at 562 nm, the model parameters were calculated using regression analysis. The Equations (1) and (2) were determined using the SPSS (IBM, Armonk, NY, USA). As the quadratic and linear models are approximations, the lower limit of detection was mathematically extended to 0 μg/mL and negative results at correspondingly low extinctions were set to 0.

Protein concentration = −6.8 + 908.0 × sample + 47.3 × sample^2^ (1)

Protein concentration = 1.77 + 427.7 × sample(2)

### 3.1. Descriptive Statistics for Protein Concentrations before and after Treatment, as Well as Difference (Increase or Decrease)

**Figure 3:**
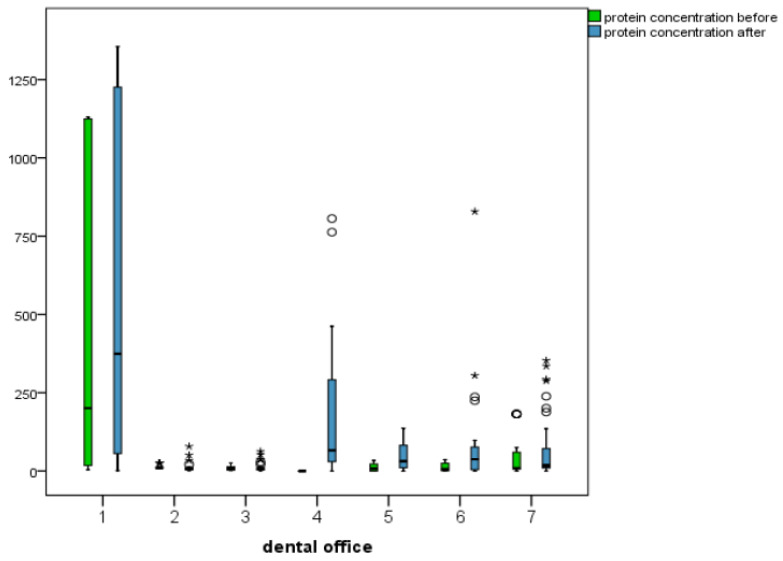
Protein concentrations before and after treatment for each dental office (* and ° for outliers).

The protein concentrations of 363 out of 398 obtained samples (91.2%) were calculated. A change in the protein concentration compared to the initial value was found in 293 out of 304 samples (96.4%). In 208 out of 304 samples (68.4%), an increase in protein concentration was calculated. The average increase was +72.1 (standard deviation 125.2). 85 samples contained less protein after treatment, with an average decrease of −12.6 (standard deviation 24.8). Eleven samples with a starting concentration of 0 μg/mL showed no change after treatment. There was a measurable protein concentration in 75 out of 94 (79.8%) water samples collected prior to treatment. Altogether, a wide distribution of values was observed. The highest before-treatment concentration was found to be 1130.7 μg/mL, and the highest after-treatment concentration was 1356 μg/mL, both in Office One. Here, the standard deviation was also the highest. The lowest concentrations of before- and after-treatment samples were 0.0 μg/mL. The greatest decrease in concentration when compared to the value before treatment was −160.5 μg/mL. The greatest increase was 864.6 μg/mL in Office Six shown in Figure 3.

### 3.2. Final Statistics—Combined Influence of Parameters on Protein Concentration after Treatment and Difference (Increase or Decrease)

**Figure 4:**
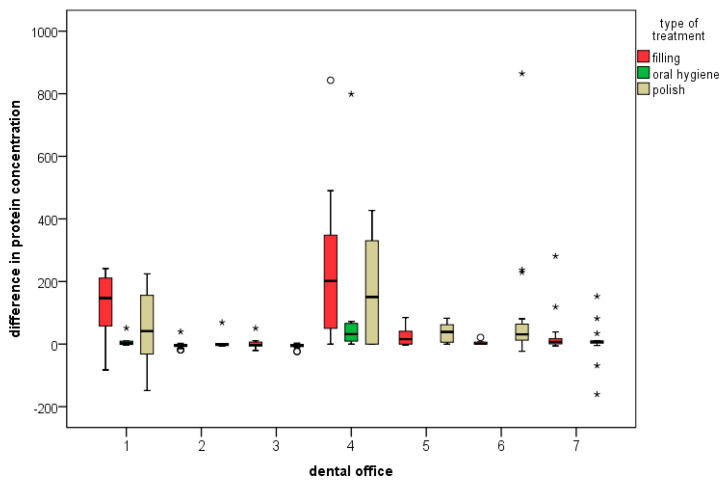
Correlation of the investigated office with the type of treatment (* and ° for outliers).

**Figure 5:**
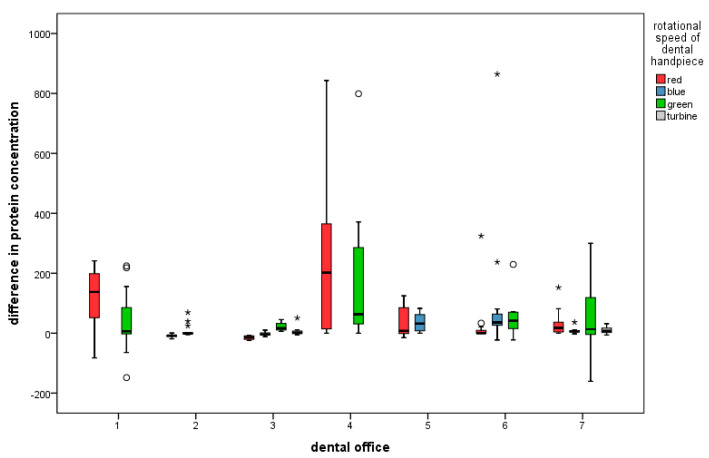
Correlation of office with the type of handpiece used (* and ° for outliers).

In determining the combined influence of the parameters using regression analysis, the office (*p* = 0.001) and the office in combination with the type of treatment (*p* = 0.004) showed a significant influence on the protein concentration after treatment (Figure 4). Only the office in combination with the rotational speed of the dental handpiece and the type of treatment showed a significant difference (*p* = 0.003) in protein concentration before and after treatment (Figure 5). Therefore, the individual work processes have the strongest influence on the protein concentration after treatment and the difference in the concentration before treatment. However, in calculating the influence of the individual parameters, the possibility of a purely coincidental result must be considered.

### 3.3. Three Consecutive Treatments per Day over the Course of Two Days in Two Dental Offices

**Figure 6:**
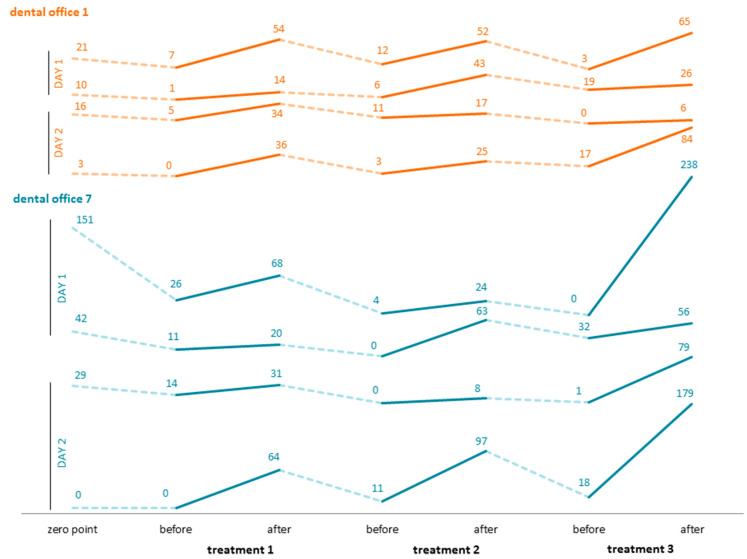
Protein concentrations over the course of two days.

As shown in Figure 6, the increase in protein concentration after treatment is consistently detectable in each of the two offices. A negative difference between the preceding after-treatment value and the subsequent before-treatment value is clearly detectable. The final values following the third treatment of the first day are higher than the starting values of the second day following internal decontamination of the instruments. The highest protein load was found to be 238 μg/mL after the third treatment in dental office 7. The lowest concentrations were 0.0 μg/mL.

## 4. Discussion

### 4.1. Outcome

In dental medicine, a wide variety of instruments lead to an unacceptable risk of cross-infection, unless they are decontaminated according to current guidelines [17]. Although the use of appropriate precautions to prevent the spread of pathogens has become routine in dentistry [36], high-speed handpieces continue to be the weak link in the chain of sterility [37,38,39]. This is due to weak spots on the inside of the instruments and mechanisms that have been identified as potential sources of infection in numerous laboratory studies. In 1992, Lewis and Boe [17] clearly showed internal contamination of instruments using food coloring applied externally to handpiece turbines before they were run for a defined amount of time. Hauman [40] also showed that though disinfection of external surfaces leads to a reduction in bacterial counts, there are bacteria on the inside despite a five-minute rinse with water. The results of the present study, geared towards everyday practice, also show that in the daily use of handpieces, water and air lines, and/or the inner surfaces of the instruments may be contaminated. This contamination most likely occurs due to the retraction of spray water when the instrument is switched off as well as due to the high-speed rotation of the drill in the chuck. In 2019, Pantanella et al. investigated the efficacy of acoustic waves in preventing *Streptococcus mutans* adhesion on dental unit water lines. Retraction of human fluid during dental therapy was found to be the contamination source. Elastic acoustic waves at high energy were deployed, preventing bacterial adhesion [41]. The fact that 26 out of 28 (92.9%) water samples taken after procedures during which no spray water was used showed an increase in protein concentration illustrates that the contamination cannot be due solely to the retraction of spray water. Altogether, a change in protein concentration was found in 293 out of 304 (96.4%) samples drawn after treatment. A study by Lewis et al. [16] illustrates the potential consequences of this technical weak point. In the study, an AIDS patient was treated with two different handpieces. Proviral HIV-DNA was shown in both. In trial series A, one handpiece was used for several treatments in the course of one working day without cleaning or disinfecting the inside surfaces of the handpiece between treatments. In this way, several before- and after-treatment samples were compared to one before-treatment value. The resulting differences in concentration—sometimes a decrease, and sometimes an increase—can indicate retraction and expulsion of infectious secretions. As the measurements of trial series B were carried out over the course of two days, the findings of trial series A were confirmed. In both offices on both days, the value of the first sample from the cleaned handpiece was higher than the value of the sample immediately prior to the first treatment, which points to a certain dilution effect. In all samples taken after treatment, higher concentrations were found than before treatment. For example, in both offices (office 1 and office 7) a higher concentration was found after the third treatment on each of the two days. The consistently lower value before the second and third treatments can also be explained by dilution. Both descriptive and inductive statistics show great differences depending on the office studied. Office 1, for example, showed the highest starting and ending concentrations, whereas Office 4 showed a steeper increase in protein concentration. It is conspicuous that the samples from the turbines, i.e., the instruments with the highest rotational speed, showed the lowest protein concentrations. However, only two of the offices worked with such instruments. Michels and Schulz-Fincke [42], on the other hand, found higher contamination in turbines than in handpieces. Most outliers for the after-treatment samples were found for the red handpiece that was used in all offices. However, no statement can be made that a particular type of handpiece (red angular piece, turbine, etc.) is more or less exposed to proteins based on its function. In order to be able to make a conclusive statement in this regard, further studies would have to be undertaken with greater numbers, brand-new instruments, and less variability in application. In addition, the producing company was recorded only for purposes of documentation, and no conclusions concerning advantages or disadvantages can be drawn. The formation of aerosols during dental treatment with dental high-speed instruments and ultrasonic scaling is a topic of attention [43,44,45,46,47,48]. A recently published study investigated the effect of ultrasonic scaling on aerosol formation and cross-contamination. In a double-masked two-group clinical trial, 10 healthy subjects were divided into two groups, which underwent preprocedural mouth rinsing with essential oil, povidone-iodine, or water. Bacterial contamination was reduced with preprocedural mouth rinsing, depending on the applied procedure [49]. In 2021 Farah et al. examined the effect of adding food-grade thickeners to the coolant solution to prevent the formation of aerosols during ultrasonic scaling and therefore prevent cross infections. The vertical spread of contaminated splatter was reduced by 80% compared to the control group [50]. The regression analysis of the protein concentrations after treatment shows a significant influence for the office (*p* = 0.001) and for the office in combination with the type of treatment (*p* = 0.004). Concerning the difference in protein concentrations before and after treatment, the office in combination with the type of treatment and the rotational speed of the handpiece was significant with *p* = 0.003. As these treatments could not be standardized, the time for which the instrument was used at maximum rotational speed could not be considered and the correlation with a particular handpiece may thus be coincidental. Regardless, the extent of contamination can depend on the person using the instrument as well as on the patient. Additional relevant factors include the number of motor stops, the rotational speed of the handpiece (controlled using the foot pedal), the extent of the lesion to be treated, and the oral hygiene of the patient. The initial values of the water samples obtained from the freshly decontaminated instruments in both trials must especially be stressed. In 75 out of a total of 94 (79.8%) water samples obtained before treatment, a protein concentration was calculated. Thus, six out of seven offices had contaminated spray-water lines even before patients were treated with these instruments. It is conspicuous that in the case of instruments in the office where thorough decontamination, including disinfection, had been performed, no protein was measurable before treatments. An exact documentation of the decontamination procedures and storage conditions, as well as an analysis of the disinfectants and lubricants used, could shed light on the amount of protein left after cleaning. Individual handling of the instruments for decontamination and smooth functioning is essential too. For example, when blood is denatured at higher temperatures (80 °C), fixation of blood proteins on instrument surfaces can occur [51]. Nevertheless, the records show a clear reduction of protein concentration by daily decontamination of the instruments in devices with automatic internal cleaning, and this is therefore a mandatory step in the decontamination of instruments. The use of devices with automatic internal cleaning requires additional devices, ideally a steam sterilizer class B or S [52], for the further necessary decontamination processes of disinfection or sterilization. In order to circumvent this problem, technologies have been developed that allow cleaning and disinfection to take place in a single device, with automatic programs permitting temperature regulation up to 90 °C. The effectiveness of the performance of these devices has been validated several times [53]. A crucial argument made by dentists for not using automated decontamination of handpieces is the shortened life span of the instruments. Dentists argue, without consideration for patient safety, that acquisition of the decontamination devices, their service and maintenance, as well as the wear and tear of the handpiece require costly investments. A study that tested handpieces from nine brand-name dental companies for their function in combination with repeated thermal sterilization interestingly showed that no model had qualitative advantages over any other with regard to longevity, performance, noise, eccentricity, and other parameters. All tested instruments could withstand at least 500 sterilization cycles without impairing performance when sterilizations were performed correctly and instruments were properly lubricated [54]. Finally, the risks associated with a faulty or even absent decontamination process are severe for the patient [55]. Additional costs for the prevention of infections must be accepted by dentists.

### 4.2. Limitations

The utilization of the BCA Assay for the detection of protein concentrations in high-speed dental instruments has its limitations. The reduction of Cu^2+^ ions to Cu^1+^ by proteins with subsequent chelation complexing and photometric measurement cannot directly establish a relationship between microorganisms from the biofilm inside the water lines and retracted saliva. In 2019 a study by Jiang et al. investigated the oral microbiome of elderly people with and without dental caries [55]. Microbiological examinations before and after treatment with high-speed dental handpieces would be necessary to detect potential pathogen microbiota inside the instruments. Another important factor can be the water quality itself, which can influence the biofilm formation inside the dental unit. For further investigations, samples of the provided water should be taken and examined for their microbiome. The water temperature and water stagnation times can also influence the quality of the water provided [56,57,58,59,60,61]. For this reason, physical and chemical properties as well as microbiological setup should be combined for a better understanding of the dental unit environment.

## 5. Conclusions

This study was conducted in response to the tremendous current hygienic conditions in dental offices, largely due to non-adherence to existing guidelines concerning the decontamination of dental handpieces. The results clearly prove the massive contamination, detected with the BCA Assay, of the used instruments, and the possibility of infection transmission by contaminated spray water. Therefore, after each patient’s treatment, automatic cleaning and disinfection of used handpieces using validated methods is necessary in dental offices. Automatic decontamination of the inner and outer surfaces of the handpieces is necessary for minimizing the risk of cross-contamination in the dental office. Microbiological investigations of the dental units should be performed to support the findings in this study with respect to bacterial contamination due to biofilm formation.

## Figures and Tables

**Table 1:** Dilution scheme for test tube protocol with a working range of 20–2000 µg/mL.

Vial	Volume of Diluent (µL)	Volume and Source of BSA	Final BSA Concentration (µg/mL)
A	0	300 of Stock	2000
B	125	375 of Stock	1500
C	325	325 of Stock	1000
D	175	175 of vial b	750
E	325	325 of vial c	500
F	325	325 of vial e	250
G	325	325 of vial f	125
H	400	100 of vial g	25
I	400	0	0

**Table 2:** Dilution scheme for enhanced test tube protocol with a working range of 5–250 µg/mL.

Vial	Volume of Diluent (µL)	Volume and Source of BSA	Final BSA Concentration (µg/mL)
J	700	100 of Stock	2000
K	400	400 of vial j	1500
L	450	300 of vial k	1000
M	400	400 of vial l	750
N	400	100 of vial m	500
O	400	0	250

**Table 3:** Handpieces used in the different examined dental offices (speed in rounds per minute (rpm)).

Office	4 × 10^5^ rpm	2 × 10^5^ rpm	4 × 10^4^ rpm	2 × 10^4^ rpm
1		x ^1^		x
2		x	x	
3	x	x	x	x
4		x		x
5		x	x	
6		x	x	x
7	x	x	x	x

^1^ x for handpiece in use.

**Table 4:** Decontamination methods of the individual dental offices.

	Wipe Disinfection,	Wipe Disinfection,	Wipe Disinfection
		Mech. Cleansing	Mech. Cleansing
Office			Mech. Disinfection/Sterilization
1		x ^2^	
2		x	
3	x		
4			x
5		x	
6		x	
7		x	

^2^ x for the method used in each dental office.

## Data Availability

Not applicable.
